# Construction of N-Doped Carbon-Modified Ni/SiO_2_ Catalyst Promoting Cinnamaldehyde Selective Hydrogenation

**DOI:** 10.3390/molecules28104136

**Published:** 2023-05-17

**Authors:** Yongwang Ren, Huizhong Xu, Beibei Han, Jing Xu

**Affiliations:** 1 School of Naval Architecture and Maritime, Zhejiang Ocean University, Zhoushan 316022, China; renyongwang4465@163.com; 2 SGS-CSTC Standards Technical Services Co., Ltd., Shanghai 201205, China; glen.xu@sgs.com; 3 Zhejiang Tianyuan Fabric Co., Ltd., Wenling 317513, China; 4 National Engineering Research Center for Marine Aquaculture, Zhejiang Ocean University, Zhoushan 316022, China

**Keywords:** Ni catalyst, N-doped carbon, electronic effect, cinnamaldehyde hydrogenation

## Abstract

At present, the selective hydrogenation of α, β-unsaturated aldehydes remains a challenge due to competition between unsaturated functional groups (C=C and C=O). In this study, N-doped carbon deposited on silica-supported nickel Mott–Schottky type catalysts (Ni/SiO_2_@N_x_C) was prepared for the selective hydrogenation of cinnamaldehyde (CAL) by using the respective hydrothermal method and high-temperature carbonization method. The prepared optimal Ni/SiO_2_@N_7_C catalyst achieved 98.9% conversion and 83.1% selectivity for 3-phenylpropionaldehyde (HCAL) in the selective hydrogenation reaction of CAL. By constructing the Mott–Schottky effect, the electron transfer from metallic Ni to N-doped carbon at their contact interface was promoted, and the electron transfer was demonstrated by XPS and UPS. Experimental results indicated that by modulating the electron density of metallic Ni, the catalytic hydrogenation of C=C bonds was preferentially performed to obtain higher HCAL selectivity. Meanwhile, this work also provides an effective way to design electronically adjustable type catalysts for more selective hydrogenation reactions.

## 1. Introduction

Catalytic hydrogenation reactions are of great importance for solving energy, environmental, and health problems [1,2,3]. The hydrogenation of α,β-unsaturated aldehydes is a typical representative of catalytic hydrogenation reactions [4,5]. In this reaction, the hydrogenation products, such as cinnamyl alcohol (COL by hydrogenation at the C=O bond) and hydrogenated cinnamaldehyde (HCAL by hydrogenation at the conjugated C=C bond), have been used in various fields, including the production of pharmaceutical intermediates, chemicals, perfumes, and fragrances [6,7,8]. Notably, as the hydrogenation position of a α,β-unsaturated aldehyde molecule changes, the hydrogenated products present various differences, which therefore requires the precise control of hydrogenation positions. However, the presence of multiple unsaturated bonds easily causes the competition of hydrogenation reactions, leading to a relatively low selectivity for a target product [4,8]. Therefore, it is urgent to develop highly selective catalysts that are capable of precisely controlling the catalytic hydrogenation of α,β-unsaturated aldehydes.

In general, changes in catalyst compositions and structures can importantly affect the physico–chemical properties of catalysts so as to govern the adsorption–desorption as well as the activation of reactants (CAL). As a result, the hydrogenated product (COL or HCAL) could be formed with a high selectivity. There have been various strategies to improve the catalytic hydrogenation of C=O or C=C bonds by specially fabricating catalysts [1,9]. Kim et al. [10,11] and Zheng et al. [12,13] prepared the catalysts by chelating organic ligands on the surface of the metal active sites, which markedly changed the adsorption mode of CAL on the active sites as a way to improve the selectivity of COL. Tang et al. [7,14] used metal organic frameworks (MOFs) compounds to form a spatial site barrier on the catalyst surface, which modulated the spatial orientation of the catalyst for CAL adsorption, thus allowing the C=O bond of cinnamaldehyde to contact the active sites more easily. In these studies, although the COL selectivities could be enhanced, the CAL conversions showed decreases in different degrees, which were due to the selective barrier that inhibited the mass transport of CAL. To improve the CAL conversion, the unique structure, uniform pore size distribution, and large surface area of mesoporous silica materials were utilized by Yang et al. [15]. The prepared mesoporous silica-supported Pd catalyst exhibited high hydrogenation activity to cinnamaldehyde, which was more than two times a commercial Pd/C catalyst. The result indicated that the mesoporous support with an excellent physical structure played an important role in promoting the catalytic activity.

Apart from the effect of catalyst structures, the electronic property of catalysts also poses a critical role in catalytic performance. In recent years, N-doped carbon-supported transition metal catalysts have been developed for catalytic reactions and have shown excellent activities in oxidation reaction (OER) [16,17], oxygen reduction reaction (ORR) [18,19], CO_2_ electroreduction reaction [20], photocatalytic reaction [21], and catalytic hydrogenation [22,23,24,25]. Moreover, our previous studies also further demonstrated that N-doped carbon-supported non-precious metal catalysts used for selective hydrogenation reactions achieved excellent catalytic activities and selectivities [24,26,27].

Zhao et al. [1] reported N-doped carbon-supported Fe–Co alloy nanoparticle catalysts for CAL selective hydrogenation. Regulating the electron density of metallic active sites could effectively modulate the product selectivity in a hydrogenation reaction. The COL selectivity was up to 91.7% together with a high CAL conversion at 95.1%. In addition, Chen et al. [28] prepared Pd@MOF (N-doped carbon material) catalysts. The introduction of nitrogen, which changed the electronic structure of Pd nanoparticles, effectively regulated the catalytic hydrogenation of CAL, leading to a 100% yield of HCAL. However, the aforementioned strategies, because of the tedious preparation process and high cost, will encounter difficulties in carrying out a large-scale industrial application. Therefore, developing non-precious metal catalysts for CAL selective hydrogenation presents a large potential. Liu et al. [29] designed N-doped carbon-covered metal Co catalysts with core–shell structures, which utilized the coordination between Co nanoparticles and N atoms, enabling Co@NC-900 to attain 99% COL selectivity. However, the application of non-precious metal catalysts in the selective conversion to HCAL remains a challenge.

In this work, silica-loaded metallic nickel modified by N-doped carbon was prepared for the selective hydrogenation of CAL to HCAL. In the synthesis of catalysts, the Ni/SiO_2_ was first synthesized by using the hydrothermal synthesis method and then the high-temperature pyrolysis to construct Mott–Schottky-type Ni/SiO_2_@N_x_C catalysts. In the Ni/SiO_2_@N_x_C catalysts, a strong electronic interaction was formed between the metallic Ni and the N-doped carbon, consequently giving rise to the Mott–Schottky effect [30]. Because of the low work function of metallic Ni as that of N-doped carbon, the peripheral electrons of metallic Ni were transferred to N-doped carbon, hence changing the charge density of metallic Ni [31]. By adjusting the N content, the electron transfer of metallic Ni could be tuned, by which the catalytic performance was promoted. The Ni/SiO_2_@N_7_C catalyst that exhibited the Mott–Schottky effect showed excellent catalytic activity, with 98.9% CAL conversion and 83.1% HCAL selectivity being obtained in CAL selective hydrogenation.

## 2. Results

### 2.1. Structure and Morphology of Ni/SiO_2_@N_x_C

**Figure 1:**
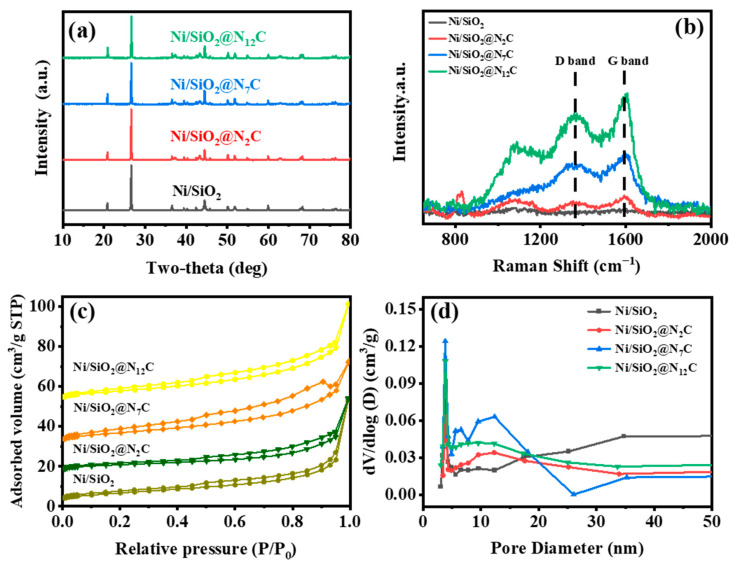
(**a**) XRD patterns; (**b**) Raman spectra; (**c**) N_2_ adsorption–desorption isotherms; and (**d**) pore size distribution curves of different samples.

The XRD patterns of the Ni/SiO_2_, Ni/SiO_2_@N_2_C, Ni/SiO_2_@N_7_C, and Ni/SiO_2_@N_12_C samples are shown in Figure 1a. It is obvious that all these XRD profiles display 3 characteristic peaks at approximately 20.8°, 26.6°, and 50.1°, which can be assigned to the (100), (101), and (112) crystal planes of silica (ICDD PDF#46-1045), respectively. In addition, 3 distinct diffraction peaks at 44.4°, 51.8°, and 76.3° corresponding to the (111), (200), and (220) crystal planes of metallic Ni were also observed, indicating the Ni-based species were reduced to metallic Ni by carbon during carbonization (ICDD PDF#87-0712). In addition, there are diffraction peaks located at 37.2°, 43.2°, and 62.8°, which correspond to the (111), (200), and (220) crystal planes of NiO (ICDD PDF#73-1523), respectively. This may be due to inadequate reduction during the high-temperature carbonization process. However, the XRD results can still indicate that the nickel-based species were reduced in situ to metallic nickel by the carbon-reducing agent during the high-temperature pyrolysis process. To illustrate the fact that the Ni species were reduced to Ni^0^ by carbon, Raman spectra were carried out to give further proof, as shown in Figure 1b. As expected, the Raman profiles exhibited 2 typical characteristic peaks of carbon material, assigned to the D band at 1340 cm^−1^ and G band at 1590 cm^−1^ [26,32], respectively. The results indicated that the carbon residues which were originally from polydopamine pyrolysis and therefore led to the reduction of the Ni species to Ni^0^ were deposited on Ni/SiO_2_. The BET surface area and pore structure of various samples were obtained by N_2_ physical adsorption. Figure 1c exhibits the N_2_ adsorption–desorption isotherms, on which obvious hysteresis loops were detected. This indicated that mesopores were constructed on these as-prepared samples. The mesopore sizes for all of the samples were focused on 13 nm, as shown in Figure 1d. As displayed in Table 1, the BET surface areas of samples were almost indistinguishable from 15.78 m^2^/g (Ni/SiO_2_) to 22.23 m^2^/g (Ni/SiO_2_@N_2_C), and the pore volume was in a range of 0.059–0.078 cm^3^/g. The formed mesoporous in catalysts is a prerequisite for effectively performing the catalytic reaction.

**Figure 2:**
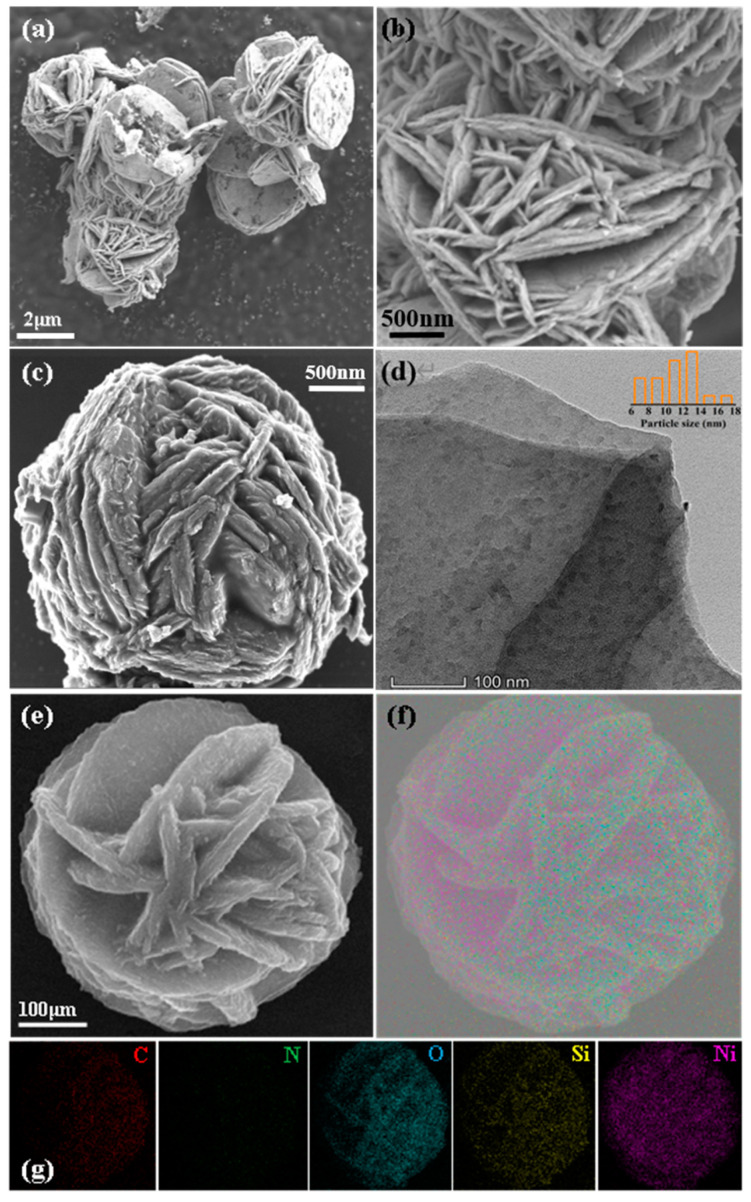
(**a**,**b**) SEM image of Ni/SiO_2_ support; (**c**) SEM; (**d**) HRTEM; (**e**) STEM-HAADF; and (**f**,**g**) element mapping images of Ni/SiO_2_@N_7_C.

The SEM images of Ni/SiO_2_ are shown in Figure 2a,b. Clearly, the Ni/SiO_2_ was stacked by thin disks, where the large amount of gaps caused by stacking could promote excellent facilitation for the mass transfer of reactant molecules. When carbon was deposited on Ni/SiO_2_, the structure and morphology was still maintained, as shown in Figure 2c. Furthermore, after carbon deposition, the surface of Ni/SiO_2_ became more wrinkled. The STEM-HAADF image is shown in Figure 2e, on which the element mapping was performed. The element mapping was also employed to reveal the element composition. As shown in Figure 2f,g, carbon and nitrogen were captured, demonstrating that the N-doped C element was successfully introduced into the catalyst, which was in agreement with the Raman result. Based on the element mapping, it was found that C and N were highly dispersed on Ni/SiO_2_ consisting of Ni, O, and Si elements. A TEM image was filmed on the Ni/SiO_2_@N_7_C sample, as shown in Figure 2d. The Ni nanoparticles present a high dispersion on the SiO_2_ support, with particle sizes at 12–14 nm. These sizes are similar to that (15.6 nm) calculated by the Scherrer formula. In light of the covering of N-doped carbon on Ni/SiO_2_, it could be imagined that an interaction was formed between metallic Ni and N-doped carbon. Their relationship is described in detail in the following content.

The above observation and analysis demonstrated that the N-doped carbon was successfully deposited on Ni/SiO_2_. After introduction, the N-doped carbon not only maintained the original disk structure, however also provided the possibility of the construction of the Mott–Schottky effect caused by electron transfers between metallic Ni and deposited N-doped carbon.

**Figure 3:**
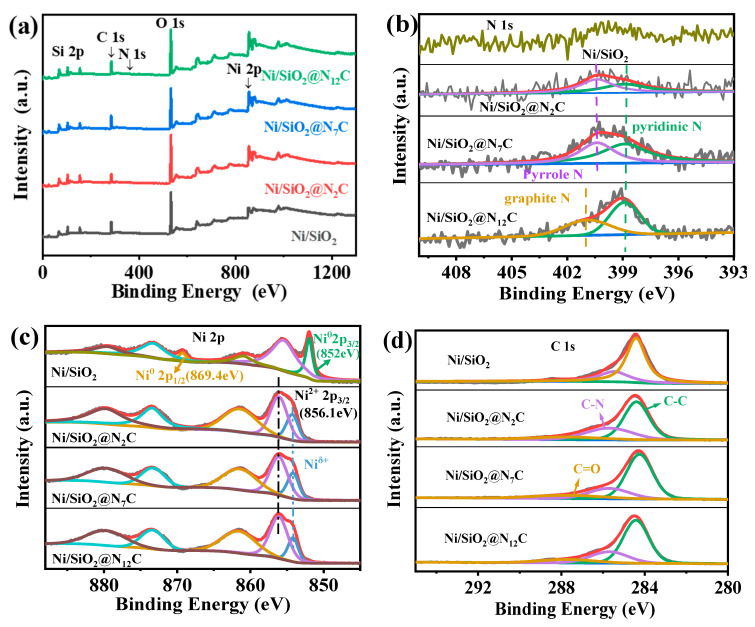
(**a**) XPS survey; (**b**) XPS Ni 2p spectra; (**c**) XPS N 1s spectra; and (**d**) XPS C 1s spectra of different samples.

XPS spectra were used to analyze the elements’ valency and the local electronic environment of the metallic Ni in different samples. The XPS full spectrum results (Figure 3a) show that the elements C, N, Si, O, and Ni were detected in all Ni/SiO_2_@N_x_C samples, according to element mapping analysis. The N 1s spectra of samples were deconvoluted, and 2 N 1s peaks were observed on the Ni/SiO_2_@N_2_C and Ni/SiO_2_@N_7_C samples in Figure 3c. The peak at approximately 398.9 eV belongs to pyridinic N, and the other peak at approximately 400.4 eV were ascribed to pyrrole N [1,33,34]. In contrast, the characteristic peaks of pyridinic N (398.9 eV) and graphitic N (400.9 eV) were observed in Ni/SiO_2_@N_12_C. For N-doped carbon materials, it has been generally accepted that the introduction of pyridine N into the carbon skeleton could enhance the electronic interaction with the active metal and thus improve the catalytic performance [35]. Our previous studies also affirmed that the doping of pyridine N could increase the electronic interactions of carbon and supported metals, because the chemical inertness of carbon could be activated by N doping [25,35]. In this work, Ni/SiO_2_@N_x_C, due to the introduction of N-doped carbon, indeed exhibited a different catalytic result over Ni/SiO_2_ for the selective hydrogenation of cinnamaldehyde. Combined with the results of the N 1s spectra, the Ni 2p spectra of all samples were performed to identify the electronic structure of metallic Ni. As shown in Figure 3b, the high-resolution Ni 2p spectra were obtained by Gaussian fitting. According to the fitting results, the peaks appearing around binding energies 852 eV and 869.4 eV for the Ni/SiO_2_ catalysts without N-doped carbon introduction are designated as Ni 2p_3/2_ and Ni 2p_1/2_ [36,37], together with the peak at 856.1 eV corresponding to Ni^2+^; the Ni^2+^ species generated was mainly due to the partial oxidation of surface Ni^0^ [38]. However, unlike Ni/SiO_2_, the Ni/SiO_2_@N_x_C series catalysts all show a characteristic peak at 854.2 eV in the Ni 2p spectra, which is attributed to Ni^δ+^. The appearance of this peak is identified as a result of the Mott–Schottky effect. It also provides evidence of the phenomenon that the electrons of metallic Ni might be shifted to the N-doped carbon. The C 1s XPS spectra of samples were also fitted by a Gaussian fitting curve. After deconvolution, 3 signal peaks with binding energy at 284.6 eV, 285.7 eV, and 287.7 eV were retrieved, which are related to C-C, C-N, and C=O [39,40,41] species, respectively, in Figure 3d. The presence of C-N indicates that N species are incorporated into the carbon skeleton to form pyridine N, which is confirmed by the N 1s spectra.

### 2.2. Catalytic Reactions

#### 2.2.1. Catalytic Performance of Catalysts

**Figure 4:**
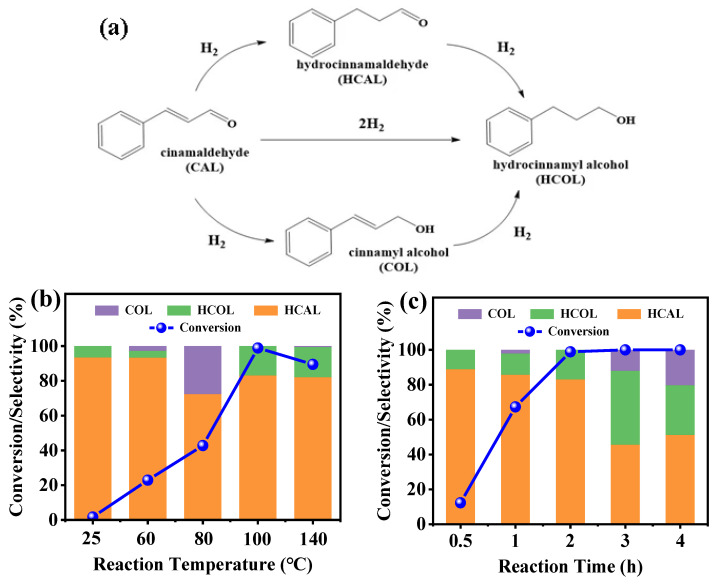
(**a**) reaction schematic and (**b**) effect of reaction temperature on CAL catalytic hydrogenation over Ni/SiO_2_@N_7_C catalyst. Reaction conditions: 50 μL cinnamaldehyde, 50 mg catalyst, 6 mL ethanol, 2 MPa H_2_, and reaction time of 2 h, and (**c**) effect of reaction time on CAL catalytic hydrogenation over Ni/SiO_2_@N_7_C catalyst. Reaction conditions: 50 μL cinnamaldehyde, 50 mg catalyst, 6 mL ethanol, 2 MPa H_2_, and reaction temperature 100 °C.

As shown in Figure 4a, the hydrogenation of CAL could occur at C=O and C=C bonds, leading to the formation of cinnamyl alcohol (COL), hydrogenated cinnamaldehyde (HCAL), and hydrogenated cinnamyl alcohol (HCOL), respectively. In this work, the Ni/SiO_2_@N_x_C catalysts were tested for the hydrogenation of cinnamaldehyde. As shown in Table 2, the conversion of CAL in the absence of a catalyst was only 0.35% (entry 1). When Ni/SiO_2_ was used, the conversion of CAL was up to 100%; however, unfortunately, the selectivity to HCAL was only 2.1% (entry 2). Compared with the Ni/SiO_2_ catalyst, the Ni/SiO_2_@N_x_C catalysts fabricated by constructing the Mott–Schottky effect exhibited good catalytic selectivity to HCAL (entries 3–5). The Ni/SiO_2_@N_2_C and Ni/SiO_2_@N_7_C catalysts obtained high cinnamaldehyde conversions at 78.3% and 98.9%, along with high HCAL selectivities at 83.7% and 83.1%. However, with the increase of N-doped carbon content in the Ni/SiO_2_@N_12_C catalyst, the cinnamaldehyde conversion decreased to 6.05% while maintaining HCAL selectivity at 76.7%. This might be related to the high content of N-doped carbon which covered a part of the Ni active sites and thus lowered the catalytic activity. Based on the above reaction results, it appears that the introduction of N-doped carbon could remarkably promote the catalytic activity of metallic Ni within a reasonable N-doped carbon content, while effectively modulating the selectivity to HCAL (enhancing the selectivity to C=C hydrogenation). The effect of reaction temperature on catalytic performance was also investigated under 2 MPa H_2_ pressure for 2 h (Figure 4b). The conversion of CAL was increased from 1.7% to 98% as the temperature was raised from room temperature to 100 °C. In relatively low temperatures, the hydrogenation of C=C bonds by the representative Ni/SiO_2_@N_7_C catalyst was more favorable than that of C=O bonds, especially. Beyond 100 °C, the selectivity of the HCAL still reached 80%. However, the product COL was over-hydrogenated to produce HCOL. Such a phenomenon suggests that the rapidly decreasing COL selectivity was due to the hydrogenation on both C=O and C=C bonds at a higher temperature. The dependence of the reaction time was investigated at 100 °C and 2 MPa H_2_ pressure (Figure 4c). An increase in the reaction time from 0.5 to 2 h led to the gradually increased CAL conversion up to 98.9%. However, the selectivity of HCAL showed a decrease after a 2 h reaction, from 83.1% to 51.2%. In addition, for comparison, other supported metal catalysts (Co/SiO_2_@N_7_C Cu/SiO_2_@N_7_C and Fe/SiO_2_@N_7_C) were also tested (entries 6–8) for CAL hydrogenation. Their catalytic activities were far below our expectations. Although the Fe/SiO_2_@N_7_C catalyst achieved relatively high HCAL selectivity compared to commercial Pd/C catalysts, the unacceptable fact was that the former only attained a 2.74% CAL conversion. In addition, the effect of different solvents on the selective hydrogenation of CAL was investigated in this work, and surprisingly, the Ni/SiO_2_@N_7_C catalyst achieved high HCAL selectivity in all organic solvents except n-hexane. Among them, when water was the solvent, its nearly 100% HCOL selectivity was astounding, although the conversion of CAL was only approximately 70%. In summary, this catalytic system has wide applicability to more hydrogenation models as well as reaction solvents.

#### 2.2.2. Construction of Mott–Schottky Effect on the Catalytic Performance of Ni/SiO_2_@N_7_C Catalysts

Considering the significant differences in the catalytic performances of the Ni/SiO_2_ and Ni/SiO_2_@N_7_C catalysts, it might be due to the introduction of the N-doped carbon that improved the catalytic performance of metallic Ni for the selective hydrogenation of cinnamaldehyde. Although the underlying reason was difficult to be revealed, this might be due to the electronic interaction formed between Ni and N-doped carbon. The accepted view in the catalytic activity was that the electron density of 3d orbitals of metallic Ni was importantly changed, as it played a key role in the adsorption of reactant molecules and subsequent activation to form active species [25,42,43]. In the present work, N-doped carbon deposited on Ni/SiO_2_ was prepared by high-temperature pyrolysis by dopamine self-polymerization. A consensus has been reached that the introduction of N not only improved the electronic density of the states of carbon but also changed the electrical conductivity of carbon [44,45,46]. As a result, an interfacial effect (Mott–Schottky effect) between the N-doped carbon and metallic nickel was induced, which led to an electron transfer between these 2 heterogeneous medias and thus changed the electron density of 3d orbitals in metallic nickel [43].

**Figure 5:**
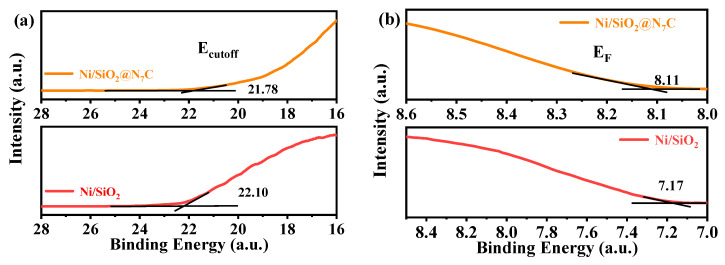

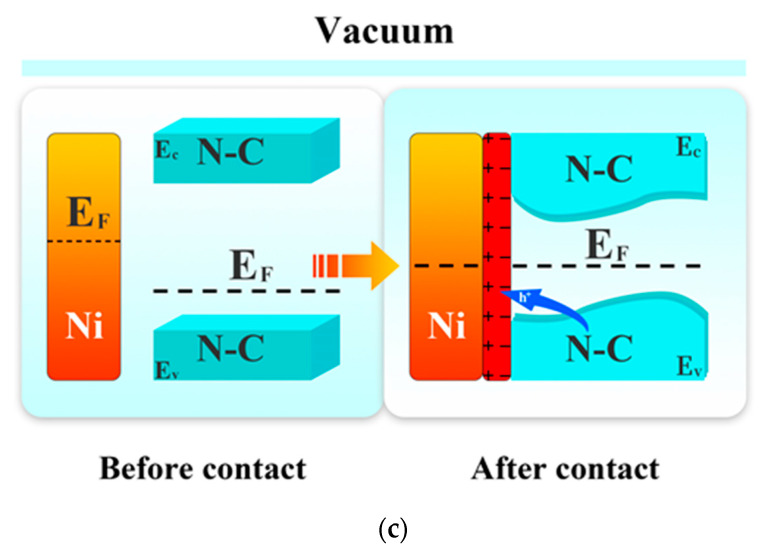
(**a**,**b**) UPS spectra of Ni/SiO_2_ and Ni/SiO_2_@N_7_C samples (Note: E_cut off_ and E_F_ represent the low energy cutoff edge and the Fermi energy level, respectively); and (**c**) The Mott–Schottky contacting interface of metallic Ni and N-doped carbon of Ni/SiO_2_@N_7_C.

To verify this phenomenon of electron transfer, we employed UPS spectroscopy to measure the work function of Ni/SiO_2_ and Ni/SiO_2_@N_7_C, as shown in Figure 5a,b. The work function has been defined loosely as the minimum energy required to extract one electron from a metal. In other words, the work function can be simply described as the ability of possessing or capturing an electron over an atom. In order to obtain the electronic structure information, the work function was determined by Einstein photoelectric law (Φ = hv − (*E_cut off_* − *E_F_*), hv = 21.21 eV), where hv, *E_cut off_*, and *E_F_* represent the incident photon energy, the low kinetic energy cutoff edge, and the Fermi energy level, respectively.

Figure 5a,b shows the UPS spectra, and the work functions of Ni/SiO_2_ and Ni/SiO_2_@N_7_C were determined to be 7.54 eV and 6.28 eV, respectively. Clearly, the introduction of N-doped carbon caused a significant change in the energy band structure of metallic Ni. Since the work function of N-doped carbon material is higher than metallic Ni, the latter inevitably transferred electrons to the N-doped material until their Fermi levels achieved an equilibrium. This subsequently led to a decrease in the electron density of metallic Ni and the formation of partially positively charged Ni^δ+^. For a better understanding of the Mott–Schottky contact, Figure 5c shows the situation before and after the contact between metallic Ni and N-doped carbon. N-doped carbon is considered a P-type semiconductor material [25,32]. As demonstrated by references, the catalytic reaction was most likely to occur at the contact interface [26]. It is also due to the controllability of metallic Ni interfacial charges that both catalytic activity and selectivity could be promoted by tuning the electronic structure. With the introduction of nitrogen-doped carbon in the Ni/SiO_2_ catalyst, the typical XPS peak of Ni (Figure 4a) gradually shifted to the higher binding energy. Combined with XPS and UPS analysis results along with catalytic activities, the N-doped carbon indeed induced the electron transfers of metallic Ni, consequently constructing the Mott–Schottky effect and improving the catalytic performance of metallic Ni.

### 2.3. Catalytic Stability

**Figure 6:**
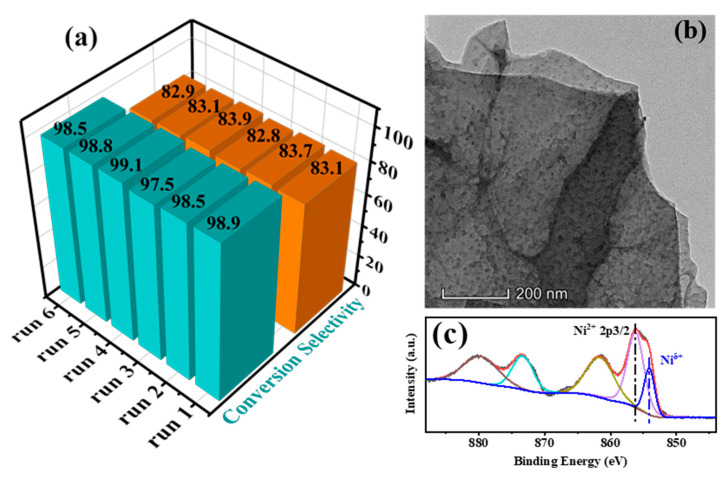
(**a**) recycling tests of selective hydrogenation of cinnamaldehyde over Ni/SiO_2_@N_7_C catalyst; (**b**) HRTEM image; and (**c**) XPS Ni 2p spectra of the used samples. Reaction conditions: 50 μL CAL, 50 mg catalyst, 6 mL ethanol as solvent, 2 MPa H_2_, reaction temperature 100 °C, and reaction time 2 h.

In heterogeneous catalytic reaction systems, the stability of catalysts is an important indicator of catalyst performance. Therefore, the catalytic stability of the representative Ni/SiO_2_@N_7_C catalyst was examined. After each run of the reaction, the sample was recovered by centrifugation to remove the reaction substances. Subsequently, the recovered sample was washed twice with water and ethanol to remove substrate residues and dried under a vacuum at 80 °C for 12 h before the next cycle. The stability test showed that the conversion of CAL and the selectivity of HCAL remained almost unchanged by the six repeated experiments in Figure 6a, and there was no activity loss observed, indicating that the Ni/SiO_2_@N_7_C had a strong catalytic stability. Additionally, an HRTEM image of the used Ni/SiO_2_@N_7_C catalyst was taken. It is clear that in Figure 6b, the structure of the Ni/SiO_2_@N_7_C catalyst was well preserved after six replicate tests. As a result, the Schottky contact interface could be maintained to exert the Mott–Schottky effect, contributing to the excellent catalytic activity and catalytic stability of the Ni/SiO_2_@N_7_C catalyst. In addition, the XPS spectrum of the used Ni/SiO_2_@N_7_C was characterized as shown in Figure 6c. The XPS spectrum displayed similar photoelectron peaks as fresh Ni/SiO_2_@N_7_C. According to the peak positions, the surface metallic Ni was still mainly in the form of Ni^δ+^, meaning that a strongly electronic interaction was built between the metallic Ni and N-doped carbon. According to the characterization results, the well-maintained structure and electronic property of the Ni/SiO_2_@N_7_C catalyst contributed to the excellent catalytic stability, in which the Mott–Schottky effect stably induced the electron transfers from metallic Ni to N-doped carbon and thus kept a high catalytic selectivity to HCAL.

## 3. Materials and Methods

### 3.1. Materials

Nickel nitrate hexahydrate (Ni(NO_3_)_2_·6H_2_O), crystalline silicon dioxide (SiO_2_), nickel chloride hexahydrate (NiCl_2_·6H_2_O), cobalt nitrate hexahydrate (Co(NO_3_)_2_·6H_2_O), cupric nitrate trihydrate (Cu(NO_3_)_2_·3H_2_O), ferric nitrate nonahydrate (Fe(NO_3_)_2_·9H_2_O), ammonium chloride (NH_4_·Cl), ammonia (25 wt% NH_3_·H_2_O), and tris (hydroxymethyl) aminomethane (C_4_H_11_NO_3_) for catalyst preparation were purchased from Sinopharm Chemical Reagent Co., Ltd., Shanghai, China. Dopamine hydrochloride (C_8_H_11_NO_2_-HCl) was provided by Shanghai Aladdin Bio-Chem Technology Co., Ltd., Shanghai, China.

### 3.2. Catalyst Preparation

#### 3.2.1. Preparation of Ni/SiO_2_ Catalyst

A total of 0.2 g of SiO_2_ and 40 mL of deionized water were added successively to a 200 mL beaker and sonicated for 40 min to form turbid solution A. Further, 2.7 mmol nickel chloride hexahydrate (NiCl_2_·6H_2_O) and 10.0 mmol ammonium chloride (NH_4_·Cl) were added into 40 mL of deionized water with stirring at room temperature for 5 min and then 2 mL of ammonia (NH_3_·H_2_O) was slowly added to form solution B. Subsequently, solution B was added dropwise into solution A in a rigorous stirring for 10 min. The mixed solution was transferred to a sealed, Teflon-lined autoclave for hydrothermal synthesis at 120 °C for 20 h. Finally, the light green solid was separated by centrifugation, washed with deionized water, and dried at 100 °C, followed by calcination at 500 °C for 2 h.

#### 3.2.2. Preparation of Ni/SiO_2_@N_x_C Series Catalysts

The N-doped, carbon-covered Ni/SiO_2_ catalysts were prepared by a high-temperature carbonization method using polydopamine as a carbon source. A tris buffer solution was firstly prepared by dissolving 0.112 g of hydroxymethyl aminomethane in 50 mL of deionized water under vigorous stirring. After this, the Ni/SiO_2_ (2.5 g) and dopamine hydrochloride (1.425 g) were added to the as-prepared solution under stirring for 3 h. The effect of varying the amount of dopamine used in the preparation was also studied (0.365 g; 2.138 g). Subsequently, it was washed twice with deionized water and alcohol, respectively. The obtained solid was dried overnight at a temperature of 80 °C. Further, the resulting solid was carbonized in an N_2_ atmosphere at 700 °C for 2 h (with a temperature rise rate of 10 °C/min). The prepared catalysts were named Ni/SiO_2_@NxC (x represents the N molar amount in dopamine). The loading amount of metal Ni for Ni/SiO_2_@N_x_C catalysts was calculated at approximately 10 wt%, as determined by inductively coupled plasma–atomic emission spectroscopy (ICP–AES).

#### 3.2.3. Preparation of M/SiO_2_@N_7_C Series Catalysts

The procedure for the preparation of the N-doped carbon-covered M/SiO_2_@N_7_C catalysts is the same as that of a Ni/SiO_2_@N_7_C catalyst, except for the replacement of NiCl_2_·6H_2_O with Co(NO_3_)_2_·6H_2_O, Cu(NO_3_)_2_·3H_2_O, and Fe(NO_3_)_2_·9H_2_O. In addition, the loading amounts of metal Co, Cu, and Fe for M/SiO_2_@N_x_C catalysts were calculated to be at approximately 9.2 wt%, 10.7 wt%, and 10.3 wt%, respectively, as determined by ICP–AES.

### 3.3. Catalyst Characterization

Powder X-ray diffraction (XRD) patterns were recorded on a DX-2700 X-ray diffractometer with a Cu Kα radiation source operating at 40 kV and 25 mA. Diffraction patterns were collected over a 2-theta range of 10~80° at a scan speed of 2° min^−1^. N_2_ low-temperature physisorption was carried out to measure the specific surface area and pore structure of samples on a Quantachrome Nova 2200e instrument. Prior to the test, the samples were outgassed under a vacuum condition at 200 °C for 3 h. The specific surface area and pore structure were acquired by the BET method and BJH method, respectively.

X-ray photoelectron spectroscopy (XPS) was carried out using an ESCALAB 250Xi spectrometer equipped with a monochromatic Al Kα X-ray source (1486.8 eV) to characterize the chemical state of the elements on the surface of the sample. All spectra obtained were referenced to C 1s peaks of the surface adventitious carbon at 284.8 eV. Ultraviolet photoelectron spectroscopy (UPS) was performed using a VG Scienta R4000 analyzer with a helium I irradiation light source at 21.21 eV to measure the work function of the samples.

The transmission electron microscopy (TEM) images along with scanning transmission electron microscopy images with a high-angle annular dark field scanning pattern (STEM-HAADF) were taken on a JEM-2100 (JEOL). Prior to the measurement, the sample was dispersed in ethanol by ultrasound, and the resulting suspension was then dropped onto a copper grid coated with a carbon film. The extent of defects in samples was tested by Raman spectroscopy on a HORIBA Scientific LabRAM HR Evolution spectrometer (HORIBA Jobin Yvon, Longjumeau, France) with a Peltier-cooled CCD detector and a laser wavelength of 514 nm. The loading amount of metal Ni, Co, Cu, and Fe for samples were measured with a Thermo Scientific iCAP 6000 ICP-OES analyzer.

### 3.4. Activity Evaluation

The hydrogenation reaction of CAL was carried out in a sealed, Teflon-lined stainless steel automatic high-pressure autoclave (20 mL). In a representative experiment, 50 mg of the catalyst, 50 μL of CAL, and 6 mL of absolute alcohol were placed into the autoclave. Following this, hydrogen was first introduced to remove the air 3 times and finally was charged to 2 MPa. Afterwards, the autoclave was heated to 100 °C for the hydrogenation reaction. After a 2 h reaction, the resulting liquid product was analyzed by gas chromatography equipped with a HP-5 MS column. It should be noted that prior to utilization, the Ni/SiO_2_ catalyst needed to be reduced by hydrogen at 500 °C for 2 h. The conversion and selectivity of the target product were calculated by the following formulas, respectively:

Conversion (%) = [CAL feed (mol) − CAL residue (mol)] × 100%/CAL feed (mol)  Selectivity (%) = target product (mol) × 100%/[CAL feed (mol) − CAL residue (mol)]

## 4. Conclusions

In summary, a SiO_2_-supported Ni catalyst precursor was prepared by the hydrothermal synthesis method, which was supplemented by high-temperature carbonization to prepare N-doped carbon-modified Ni/SiO_2_ Mott–Schottky-type catalysts. During the catalyst preparation, polymerized polydopamine was used as the carbon and nitrogen sources, and the Ni-based metal oxides were in situ reduced to the metallic state during the high-temperature carbonization. The simultaneously-formed nitrogen-doped carbon not only protected the Ni nanoparticles from oxidation and deactivation, however also induced the Mott–Schottky effect on the contact surface of the metallic Ni nanoparticles and the N-doped carbon. The N-doped carbon viewed as a P-type semiconductor induced the Mott–Schottky effect with metallic Ni, which effectively tuned the electronic structure of metallic Ni due to the electron transfers of metallic Ni. In the CAL hydrogenation, the introduction of N-doped carbon significantly improved the selective catalytic hydrogenation of Ni/SiO_2_ to generate HCAL. The Ni/SiO_2_@N_7_C catalyst exhibited a high 98.9% conversion of CAL and 83.1% selectivity of HCAL, much higher than that over the Ni/SiO_2_ catalyst.

## Figures and Tables

**Table 1:** The textural properties of various samples.

Samples	BET (m^2^/g)	Pore Volume (cm^3^/g)	Average Pore Size (nm) ^a^
Ni/SiO_2_	15.78	0.059	14.83
Ni/SiO_2_@N_2_C	22.23	0.064	11.55
Ni/SiO_2_@N_7_C	16.90	0.078	18.44
Ni/SiO_2_@N_12_C	21.72	0.076	13.97

^a^ Average pore size of samples were calculated with BJH method.

**Table 2:** Catalytic hydrogenation of cinnamaldehyde over different catalysts.

Entry	Catalyst	Solvent	CAL (Conv. %)	(Sel. %)		
				HCAL	HCOL	COL
1	Blank	Ethanol	0.35			
2	Ni/SiO_2_	Ethanol	100	2.1	97.9	0
3	Ni/SiO_2_@N_2_C	Ethanol	78.3	83.7	14.2	2.1
4	Ni/SiO_2_@N_7_C	Ethanol	98.9	83.1	16.9	0
5	Ni/SiO_2_@N_12_C	Ethanol	6.05	76.7	21.8	1.5
6	Co/SiO_2_@N_7_C	Ethanol	45.6	21.8	54.9	23.3
7	Cu/SiO_2_@N_7_C	Ethanol	24.8	45.6	47.8	6.6
8	Fe/SiO_2_@N_7_C	Ethanol	2.74	78.1	21.5	0.4
9	Pd/C	Ethanol	36.6	69.2	26.8	4.0
10	Ni/SiO_2_@N_7_C	Water	67.1	99.8	0	0.2
11	Ni/SiO_2_@N_7_C	Methanol	34.7	89.3	6.1	4.6
12	Ni/SiO_2_@N_7_C	n-Heptane	36.61	69.3	26.8	3.9
13	Ni/SiO_2_@N_7_C	Isopropanol	4.27	70.7	29.0	0.3
14	Ni/SiO_2_@N_7_C	n-Hexane	0.35			
15	Ni/SiO_2_@N_7_C	n-Propanol	0.59	100	0	0
16	Ni/SiO_2_@N_7_C	n-Butanol	2.54	83.5	16.5	0

Reaction conditions: 50 μL CAL; 50 mg catalyst; 6 mL ethanol; 2 MPa H_2_; reaction temperature 100 °C; reaction time 2 h.

## Data Availability

Not applicable.
